# Antipsychotic prescribing: national findings of children and adolescents attending mental health services in Ireland

**DOI:** 10.1007/s00787-024-02428-4

**Published:** 2024-04-12

**Authors:** David J. O. Driscoll, Suzanne McCarthy

**Affiliations:** 1 School of Public Health, Western Gateway Building, University College Cork, Cork, Ireland; 2 Specialist Neurodevelopmental ADHD Pathway (SNAP), Cork and Kerry Mental Health Services, Cork, Ireland; 3 School of Pharmacy, University College Cork, Cork, Ireland

**Keywords:** Off-label, Antipsychotic, Medication, Children and adolescents

## Abstract

Antipsychotic prescribing trends vary internationally, albeit off-label use remains high (i.e., target symptoms). We aim to describe antipsychotic use, target conditions, target symptoms and dosing regimens in children and adolescents in Ireland. We used a sampled cohort from a national audit of children and adolescents attending mental health services with predefined inclusion and exclusion criteria from Jul-2021 to Dec-2021 who were prescribed at least one psychotropic medication and up to and including 17-years of age (n = 3528). Each service provided anonymised data. We described the frequency of antipsychotic medication, medication type, target condition, target symptom and medication doses. We used multivariable logistic regression, adjusted with available co-variates to assess the association of being prescribed an antipsychotic medication. Twelve percentage (n = 437) were prescribed an antipsychotic and 16–17-years (n = 211, 48.3%) was the most common age category. The commonest reason for prescribing an antipsychotic was target symptoms (i.e., off-label use) (n = 329; 75.%) and of these symptoms, agitation (n = 77/329; 25%) and irritability (56/239; 25%) were the most common. Quetiapine (n = 127; 29%) was the most common antipsychotic, followed by risperidone (n = 125; 28.6%), aripiprazole (n = 107; 24.5%), and olanzapine (n = 66; 15.1%). In adjusted analysis, having a psychotic disorder ((adjusted-odds-ratio) aOR: 39.63, CI 95%, 13.40–117.22), bipolar disorder (aOR: 16.96, CI 95%, 3.60–80.00), autism spectrum disorder (aOR: 3.24, CI 95%, 2.45–4.28) or aggression symptoms (aOR: 16.75, CI 95%, 7.22–38.89) was associated with prescribing an antipsychotic medication. This is the first study in children and adolescents that describes the target conditions and target symptoms for antipsychotic use in Ireland. Our results show a high proportion of antipsychotic prescribing based on target symptoms rather than target condition or diagnosis.

**Supplementary Information:**

The online version contains supplementary material available at 10.1007/s00787-024-02428-4.

## Introduction

Approximately half of all mental health problems have an onset during or before adolescents [1, 2]. These young people require a multi-modal approach to intervention (non-pharmacological to pharmacological). A proportion of young people may benefit from a psychotropic such as an anti-psychotic medication [3, 4]. Antipsychotic medication, in particular atypical antipsychotic medications are used in the treatment of psychotic disorder, bipolar affective disorder, and Tourette’s disorder [2, 3].

Off-label or unlicensed prescribing of antipsychotic medication occurs in the paediatric population [5, 6]. Off-label prescribing is the use of medication outside of its agreed license (e.g., age of the child is lower than indicated on the license, due to indication, dose or route of administration) [6, 7]. Off-label use may be to augment treatment in diagnosable disorders (e.g., obsessive compulsive disorder) or to target a distinct symptom (e.g., irritability) or cluster of symptoms or behaviours (e.g., challenging behaviour) [4, 5]. There is some evidence of efficacy for the use of risperidone for treating aggressive behaviours in the short term, yet caution is advised by a recent Cochrane review [8, 9]. Furthermore, diagnostic practices may differ which in turn influence prescribing practices.

Prescribing an antipsychotic off-label is done at an individual clinician’s judgement, and current guidelines advise caution with consideration for medications based on side-effect profiles and patient choice [8, 9]. This is due to the lack of clear theoretical framework and limited clinical data to guide treatment. Despite this, clinicians do prescribe antipsychotic medications off-label with the aim of alleviating distress and addressing target symptoms or cluster of symptoms. From a real-time pragmatic perspective, there is a need to understand the prevalence of which antipsychotic and dose choice is prescribed for off-label target symptoms or cluster of symptoms. This is important to ensure transparency in current prescribing clinical practice, inform the need for future research in this area, and the need for a clear theoretical framework to guide clinician choice and patient safety in the paediatric population. Currently in Ireland, Aripiprazole (15 years and over) is licensed for psychosis and Aripiprazole (13 years and over) is licensed for mania in Bipolar Affective Disorder [9, 10]. The use of Risperidone, Olanzapine or Quetiapine for psychosis or mania at or under 17 years of age is off-label. Risperidone is licenced from 5 to 18 years of age for aggression in conduct disorder, or autism spectrum disorder with the caveat advice of its use to be short-term symptomatic treatment (i.e.,  < 6 weeks) [10, 11]. The use of Aripiprazole, Olanzapine or Quetiapine is off-label for non-psychotic related disorders or symptoms [12, 13].

In Ireland, mental health care for children and adolescents up to and including 17 years of age is provided by the child and adolescent mental health service (CAMHS). Each CAMHS team provides care to a designated geographical area (sectorised care), and consists of a multidisciplinary team and is led by a clinical lead consultant child and adolescent psychiatrist. CAMHS is a publicly funded service and there is limited private services in Ireland. Three main types of clinicians prescribe psychotropics for children in Ireland, e.g., general practitioner (GP), paediatricians, and child and adolescent psychiatrists. In January 2022, a report “Maskey Report” was published which highlighted several problems in the area of governance, clinical care and administrative practices in a specific CAMHS team in Ireland. This report in particular highlighted the over use of psychotropic medication with specific reference to off-label use. Due to prescribing safety concerns, a national audit of psychotropic prescribing in CAMHS was commissioned by the Irish Government. Therefore, the aim of the audit was to describe the medications prescribed, whether there was good standard of relevant prescribing care (e.g., consent, medication monitoring) and the indications for prescribed medication (by condition/diagnosis, target symptoms (i.e., off-label).

As such, using data from a nationally representative cross-sectional survey of those attending CAMHS in Ireland, the objectives of this study are: (i) to determine the prevalence of antipsychotic prescribing, (ii) to determine the conditions for which antipsychotics are prescribed, and (iii) to describe the target symptoms (i.e., off-label) being treated by antipsychotic medications.

## Methods

### Data

A national audit was commissioned in 2021 to monitor prescribing standards, medications prescribed and their indications in all community child and adolescent mental health services (multidisciplinary paediatric psychiatric services) in Ireland. This consisted of 74 paediatric psychiatric services. A total of 21 081 were attending paediatric psychiatry services in July to December 2021 and 3 528 were eligible based on inclusion criteria (i.e., attending the service, 17 years of age or under, active attendance (i.e., attending appointments and/or receiving intervention) between July 2021 and December 2021 and prescribed any psychotropic medication during the above agreed timeline) (Fig. S1). The exclusion criteria included having a moderate to severe intellectual disability, primary diagnosis of autism spectrum disorder, and not meeting above inclusion criteria. The commissioned team explained the rationale for excluding these sub-groups was that they were deserving of a separate audit due to the complex structure of care these children and adolescents require. As per the national health service in Ireland, the Health Service Executive (HSE) Clinical Audit Criteria and Guidance, if a service had greater 500 eligible participants, a sample of 10% of same was sought, and if less than 500 eligible participants, a sample of below 50 was sought. This represents 1 in 6 children attending the CAMHS services in Ireland during the study time period. Each service were asked by the commissioned team to complete a pre-piloted tool to collect data on eligible patients in their service and data was uploaded to “Smart Survey” or submitted manually. The data was uploaded by any multi-disciplinary team member albeit advised to be confirmed by the consultant child and adolescent psychiatrist in each service. Ethics was sought from the clinical research ethics committee of University College Cork by authors to complete secondary analysis of the audit data. The data controller (HSE) provided permission and released data to the authors.

### Baseline characteristics

The following baseline characteristics were obtained: gender (male, female, other), duration attending the paediatric psychiatry service (years, months), consultant in service (yes, no), age (years, months), referral type (urgent, routine), and mental disorder diagnosis provided as (yes, no) (anxiety disorder, depressive disorder, attention deficit hyperactivity disorder (ADHD) or attention deficit disorder (ADD), psychotic disorder, bipolar affective disorder (BPAD), Tourette’s or Tic disorder, eating disorder, obsessive compulsive disorder (OCD). The above listed conditions were explicitly listed in the audit tool (Table S1) and the following information was obtained from a free text “other diagnosis” section about participants: Autism spectrum disorder, oppositional/conduct symptoms, and aggressive symptoms. Data on ethnicity and socioeconomic status were not captured.

### Prescribing standards

Six prescribing standards were obtained: (1) evidence of consent documented from parent or guardian for prescribed medication (yes, no), (2) a consultant psychiatrist prescribed the medication or the decision was in consultation with a consultant psychiatrist (yes, no), (3) baseline physical monitoring was complete (yes, no, not applicable), (4) follow up physical monitoring was complete (yes, no, not applicable), (5) correspondence with participants general practitioner (family doctor) (yes, no, not applicable), (6) follow up appointment arranged and documented (yes, no, not applicable). Documenting side-effects was not recorded as a standard in the audit.

### Antipsychotic medication

The following information on antipsychotic medication was obtained: antipsychotic name, target condition, target symptoms (a target symptom was described in the absence of a target condition by the treating team), starting dose, maintenance dose, starting date of medication, and discontinuation date of medication.

### Statistical analysis

Descriptive statistics are presented as numbers and percentages for categorical variables and mean ± standard deviations for continuous variables. A two sample test of proportion was done to compare prescribing standards for psychotic and non-psychotic medication. A difference in proportion was considered significant when the p value was < 0.05. We report frequencies (%) of indication (target condition and target symptoms) if an antipsychotic was prescribed. We report starting and maintenance dose for each medication (mean, standard deviation, median and minimum to maximum). We examined available co-variates (gender, age, referral type, duration in service, no. of co-morbidities, and type of disorder/condition) associated with the likelihood of being prescribed an antipsychotic medication by using multivariable binary logistic regression, confounded with available data. Statistical analysis was completed using STATA software (v.18).

We completed three additional analyses. First, we reported frequencies (%) of indication (target condition and target symptoms) and antipsychotic prescribed (by medication type). Second, we reported starting and maintenance dose for each medication (mean, standard deviation, median and minimum to maximum) if prescribed for (a) psychotic disorder, and (b) for target symptoms. Third, we report polypharmacy if an antipsychotic was prescribed.

## Results

### Summary of demographics

**Fig. 1:**
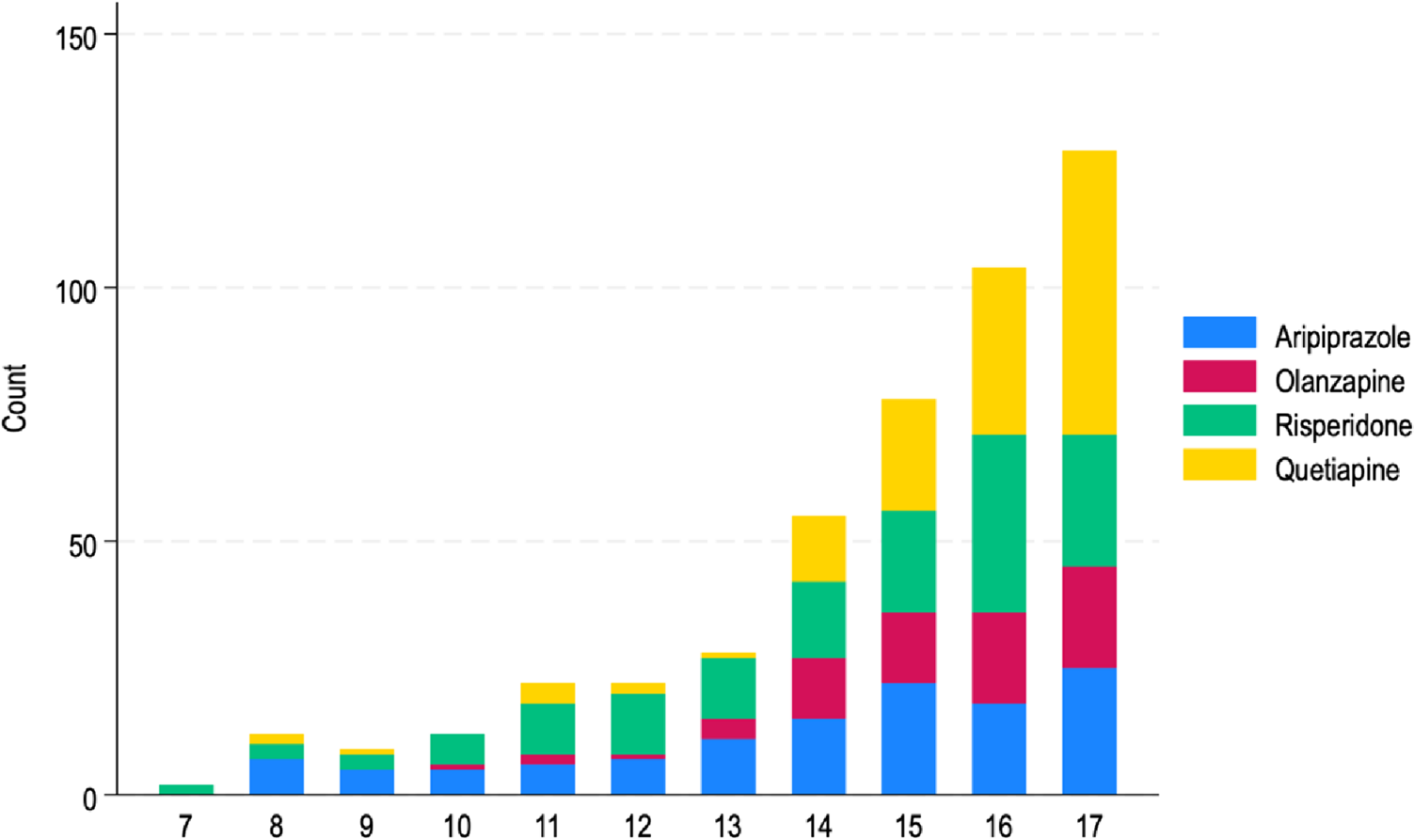
Antipsychotic type frequency prescribed by age between July 2021 to December 2021 in Ireland (n = 437)

The study sample included 3 528 children and adolescents attending the child and adolescent mental health services in Ireland between the time period July 1st, 2021, and December 31st, 2021. Over 12 percentage (n = 437) were prescribed an antipsychotic during the time period. More females (n = 231, 52.9%) were prescribed an antipsychotic, and 16–17 years was the most common age category prescribed an antipsychotic (n = 211, 48.3%) (Table 1, Fig. 1). A slightly higher proportion of children and adolescents prescribed an antipsychotic were an urgent referral to the service (n = 224, 51.3%). Forty four (n = 10.1%) of children and adolescents prescribed an antipsychotic had a psychotic disorder recorded. A higher number of psychiatric co-morbidities (n = 150, 34.3%) were reported in the antipsychotic group in comparison to no antipsychotic group (n = 564, 18.2%) The most diagnosed co-morbid disorder in children and adolescents prescribed an antipsychotic was ADD/ADHD (n = 151, 34.6%) and then anxiety disorder (n = 175, 40%). On the 31st December 2021, 70.5% (n = 2 487) of children and adolescents were prescribed one medication, 24.2% (n = 854) prescribed two medications and 5.3% (n = 187) prescribed three or four medications. The most frequent combinations with antipsychotics were: SSRI (n = 107, 3%), SSRI and melatonin, (n = 23, 0.65%) or stimulant and melatonin (n = 20, 0.57%).

**Table 1:** Summary of demographics of participants prescribed an antipsychotic and not prescribed an antipsychotic between July 2021 to December 2021 in Ireland (n = 3528)

	No Antipsychotic		Yes Antipsychotic		p value^*^
	(n = 3 091)		(n = 437)		
	n	(%)	n	(%)	
*Gender of child participant*					< 0.001
Female	1, 308	(42.3)	231	(52.9)	
Male	1, 755	(56.8)	200	(45.8)	
Other	28	(0.9)	6	(1.4)	
*Categorised duration in service*					< 0.001
< 6 months	833	(26.9)	149	(34.1)	
6–12 months	646	(20.9)	100	(22.9)	
1–2 years	502	(16.2)	64	(14.6)	
3–5 years	646	(20.9)	75	(17.2)	
6–10 years	406	(13.1)	36	(8.2)	
> 10 years	58	(1.9)	13	(3)	
*Age categorised*					< 0.001
4–7 years	43	(1)	2	(0.5)	
8–10 years	338	(10.9)	32	(7.3)	
11–13 years	740	(23)	67	(15.3)	
14–15 years	836	(27)	125	(28.6)	
16–17 years	1, 134	(36.7)	211	(48.3)	
*Referral type*					< 0.001
Routine	2, 252	(72.9)	210	(48.1)	
Urgent	820	(26.5)	224	(51.3)	
Missing	19	(0.6)	3	(0.7)	
*Moderate-Severe diagnosis*					
Anxiety disorder	894	(28.9)	175	(40)	< 0.001
ADD/ADHD disorder	1, 843	(59.6)	151	(34.6)	< 0.001
Depressive disorder	568	(18.4)	100	(22.9)	0.024
Eating disorder	130	(4.2)	63	(14.4)	< 0.001
Obsessive–compulsive disorder	144	(4.7)	23	( 5.3)	0.58
Psychotic disorder	5	(0.2)	44	(10.1)	< 0.001
BPAD	3	(0.1)	13	(3)	< 0.001
Tourette’s/Tics	30	(1)	13	(3)	< 0.001
*Number of psychiatric diagnosis*					< 0.001
1	2443	(79)	257	(58.8)	
$\ge$ 2	564	(18.2)	150	(34.3)	
*Six prescribing standards*					
Follow up arranged	3, 028	(98)	428	(97.9)	0.43
Correspondence sent to GP	2, 804	(90.7)	385	(88.1)	0.083
Baseline physical parameters prior to medication	2, 245	(72.6)	354	(81)	< 0.001
Monitor physical parameters during medication	2, 248	(72.7)	372	(85)	< 0.001
Consultant involved in prescribing	2, 941	(95.1)	428	(97.9)	0.008
Consent received to start medication	2, 764	(89.4)	387	(88.6)	0.58

*ADD* attention deficit disorder, *ADHD* attention deficit hyperactivity disorder, *BPAD* bipolar affective disorder, *GP* general practitioner (i.e., family doctor)

*Pearson’s chi-squared test, statistical significance p < 0.05

### Prescribing standards

Children and adolescents prescribed an antipsychotic medication had high prescribing standards for follow up arranged (n = 428, 97.9%), correspondence to family doctor (n = 385, 88.1%), consultant involvement in prescribing (n = 428, 97.9%) and consent obtained (n = 387, 88.6%). Baseline (n = 354, 81%) and ongoing monitoring (n = 372, 85%) of physical parameters were higher if prescribed an antipsychotic medication than not prescribed an antipsychotic medication.

### Target conditions and target symptoms of those prescribed any antipsychotic

**Fig. 2:**
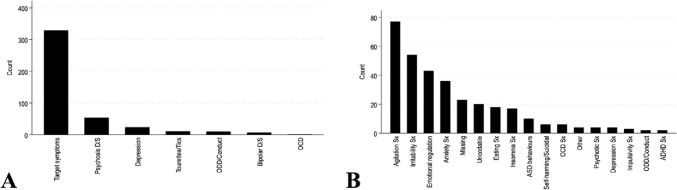
**A** Target conditions of any antipsychotic medication, **B **and target symptoms (Sx) of any antipsychotic medication. See Table 2 for (n) numbers of each medication

Of the children and adolescents prescribed an antipsychotic (n = 437), the highest target condition was not a disorder but target symptoms (n = 329, 75.5%), followed by psychotic disorder (n = 54, 12.4%) and depressive disorder (n = 24, 5.5%) (Table 2, Fig. 2A). Following breakdown of target symptoms, the highest target symptom was agitation symptoms (n = 77, 25%), irritability (n = 56, 18.2%), emotional dysregulation (n = 43, 14%) and anxiety symptoms (n = 36, 11.7%). Ten (3.2%) children and adolescents were prescribed an antipsychotic to target ASD related symptoms and behaviours. (Table 2, Fig. 2B**)** Target conditions and target symptoms are presented for each antipsychotic medication separately (Fig. S2).

**Table 2:** Description of antipsychotic prescribed target conditions (A) and target symptoms (B) between July 2021 to December 2021 in Ireland (n = 437)

(A)	Target	
Target conditions	(n = 437)	
	n	(%)
Psychotic disorder	54	(12.4)
Depressive disorder	24	(5.5)
Tourette’s/tic disorder	11	(2.5)
ODD/conduct disorder	10	(2.3)
Bipolar affective disorder	7	(1.6)
Obsessive–compulsive disorder (OCD)	1	(0.2)
Target symptoms	329	(75.5)

(B)	Target	
Breakdown of target symptoms	(n = 329)	
	n	(%)
Agitation	77	(25)
Irritability	56	(18.2)
Emotional dysregulation	43	(14)
Anxiety symptoms	36	(11.7)
Eating disorder related symptoms	18	(5.8)
Insomnia related symptoms	17	(5.5)
ASD related symptoms	10	(3.2)
OCD related symptoms	6	(1.9)
Self-harming/suicidal related symptoms	6	(1.9)
Depressive related symptoms	4	(1.3)
Psychotic related symptoms	4	(1.3)
Other	4	(1.3)
Impulsivity symptoms	3	(1)
ADHD related symptoms	2	(0.6)
ODD/Conduct related symptoms	2	(0.6)
Uncodable or missing	20	(5.8)

ODD/Conduct disorder oppositional defiance disorder/Conduct disorder, ASD autism spectrum disorder, ADHD attention deficit hyperactivity disorder. The total disorder (n = 54) in Table 1 differs to the total psychotic disorder (n = 44) in Table 2 as a participant may not have received a definitive diagnosis yet may be receiving treatment during an ongoing assessment

### Dose of antipsychotics

The most common antipsychotics prescribed were quetiapine (n = 127, 29%), risperidone (n = 125, 28.6%), aripiprazole (n = 107, 24.5%) and olanzapine (n = 66, 15.1%) (Table 3). The median starting and maintenance (s-XXmg; m-XXmg) of the antipsychotics were the following, quetiapine was s-25 mg; m-25 mg, risperidone s-0.5 mg; m-0.5 mg, aripiprazole s-2.5 mg; m-5 mg and olanzapine s-2.5 mg; m-5 mg. In sensitivity analysis, antipsychotic dose based on targeting a symptom cluster (Table 3) had a lower maintenance dose than in comparison for a psychotic disorder (Table 3).

**Table 3:** Description and breakdown of antipsychotic dose (milligrams, mg) based on: (a) all indications combined, (b) targeting a psychotic disorder, and (c) targeting symptoms only prescribed between July 2021 to December 2021 in Ireland (n = 437)

Medication	Mean	SD	Median	Min	Max
(a) Antipsychotic dose (mg) irrespective of target condition or symptom					
*Olanzapine (n = 66)*					
Starting^1^	4.7	7	2.5	1.3	50
Maintenance^2^	6.5	8.3	5	1.3	60
*Quetiapine (n = 127)*					
Starting	23.8	11.4	25	5	75
Maintenance	43.5	33	25	12.5	150
*Risperidone (n = 125)*					
Starting	0.6	0.6	0.5	0.1	5
Maintenance	0.9	0.8	0.5	0.1	6
*Aripiprazole (n = 107)*					
Starting	3.1	2.7	2.5	0.5	20
Maintenance	5.8	4.1	5	0.6	20
(b) Antipsychotic dose (mg) based on targeting a psychotic disorder					
*Olanzapine (n = 13)*					
Starting^1^	4.8	3	3.8	2.5	10
Maintenance^2^	9	4.2	10	2.5	15
*Quetiapine (n = 5)*					
Starting	25	0	25	25	25
Maintenance	100	58.6	125	25	150
*Risperidone (n = 14)*					
Starting	0.9	1	0.5	0.1	4
Maintenance	1.7	1.9	1	0.3	6
*Aripiprazole (n = 26)*					
Starting	4.3	4.6	2.5	1	20
Maintenance	8.8	4.8	7.5	2.5	20
(c) Antipsychotic dose (mg) based on target symptoms					
*Olanzapine (n = 48)*					
Starting^1^	4.8	8	2.5	1.3	50
Maintenance^2^	6.2	9.4	5.0	1.3	60
*Quetiapine (n = 107)*					
Starting	24.2	11.6	25	5	75
Maintenance	40.2	28.4	25	12.5	150
*Risperidone (n = 94)*					
Starting	0.5	0.6	0.5	0.1	5
Maintenance	0.8	0.5	0.5	0.1	3
*Aripiprazole (n = 67)*					
Starting	2.8	1.8	2.5	0.5	10
Maintenance	5.1	3.6	4.0	1.0	15

SD standard deviations

Available data (n = 425), missing or uncodable (n = 12), ^1^ Starting dose documented, ^2^ Maintenance dose documented

### Multivariable logistic regression analysis of antipsychotic prescribed

In the unadjusted model, there was a higher odds of being prescribed an antipsychotic if the child was 16–17 years ((unadjusted OR) uaOR 4.00 CI 9% 0.96–16.64), an urgent referral (uaOR 2.93 CI 95% 2.39–3.59), had an eating disorder (uaOR 3.84 CI 95% 2.79–5.28), psychotic disorder (uaOR 69.1 CI 95% 27.24–175.32), BPAD disorder (uaOR 31.56 CI 95% 8.96–111.22), ASD (uaOR 2.52 CI 95% 2.00–3.20) or aggression symptoms (uaOR 14.71 CI 95% 6.57–32.96) (Table 4).

**Table 4:** Results from logistic regression modelling of likelihood of being prescribed an antipsychotic medication

	Unadjusted^1^				Adjusted^2^			
	OR	LCI	UCI	p value	OR	LCI	UCI	p value
Gender *(ref Other)*								
Male	0.53	0.22	1.30	0.1662	0.56	0.22	1.43	0.2259
Female	0.82	0.34	2.01	0.6712	0.60	0.24	1.53	0.2846
*Age (ref 4–7 years)*								
8–10 years	2.04	0.47	8.80	0.3412	3.15	0.65	15.24	0.1538
11–13 years	1.95	0.46	8.21	0.3646	2.16	0.45	10.25	0.3331
14–15 years	3.21	0.77	13.44	0.1096	2.92	0.62	13.72	0.1749
16–17 years	4.00	0.96	16.64	0.0566	3.11	0.66	14.63	0.1506
*Referral (ref routine)*								
Urgent	2.93	2.39	3.59	0.0001	1.33	0.28	6.33	0.7195
Duration in Service	1.00	1.00	1.00	0.0353	1.00	1.00	1.00	0.0018
*No. co-morbidities*								
1	0.29	0.19	0.46	0.0001	0.30	0.14	0.67	0.0031
$\ge$ 2	0.74	0.47	1.17	0.2033	0.37	0.09	1.55	0.1749
*Disorders*								
Anxiety disorder	1.64	1.34	2.02	0.0001	0.75	0.37	1.49	0.4033
ADHD/ADD disorder	0.36	0.29	0.44	0.0001	0.40	0.20	0.81	0.0106
Depressive disorder	1.32	1.04	1.68	0.0247	0.62	0.31	1.25	0.1813
Eating disorder	3.84	2.79	5.28	0.0001	2.30	1.10	4.80	0.0269
OCD disorder	1.14	0.72	1.79	0.5778	0.62	0.28	1.38	0.2384
Psychotic disorder	69.10	27.24	175.32	0.0001	39.63	13.40	117.22	0.0001
BPAD disorder	31.56	8.96	111.22	0.0001	16.96	3.60	80.00	0.0003
Tourette’s/tic disorder	3.13	1.62	6.05	0.0007	2.28	0.88	5.95	0.0913
Autism spectrum disorder	2.52	2.00	3.20	0.0001	3.24	2.45	4.28	0.0001
Aggression symptoms	14.71	6.57	32.96	0.0001	16.75	7.22	38.89	0.0001

OR odds ratio, LCI lower confidence interval (95%), UCI upper confidence interval (95%), ADHD attention deficit hyperactivity disorder, ADD attention deficit disorder, OCD obsessive compulsive disorder, BPAD bipolar affective disorder

Odds ratio from a logistic regression model comparing the likelihood of being prescribed an antipsychotic ^1^ Unadjusted model, ^2^ Adjusted model including all covariates in table

In the adjusted model, age did not determine a higher odds of being prescribed an antipsychotic nor if the referral was urgent. There was a higher odds of being prescribed an antipsychotic if the child had an eating disorder ((adjusted OR) aOR 2.30 CI 95% 1.10–4.80), psychotic disorder (aOR 39.63 CI 95% 13.40–117.22), BPAD disorder (aOR 16.96 CI 95% 3.60–80.00), ASD (aOR 3.24 CI 95% 2.45–4.28) or aggression symptoms (aOR 16.75 CI 95% 7.22–38.89) (Table 4).

## Discussion

This is the first article using a large national sample that describes the target disorders and target symptoms of antipsychotic medication prescribed for children and adolescents attending mental health services in Ireland between July to December 2021. We illustrate that the prevalence of antipsychotic prescribing by specialist child and adolescent mental health services in Ireland is 12%, and that the main indication for prescribing an antipsychotic is to target symptom clusters and not a disorder. We also illustrate in a real time pragmatic way, the starting doses and maintenance doses that clinicians are using to target symptom clusters and psychotic disorders.

The data highlights a high amount of off-label prescribing of antipsychotic medications to young people attending specialist child and adolescent mental health services in Ireland. There is limited licensed indications for use of antipsychotics in children or adolescents in Ireland. As such, any indication based on target symptoms alone without a defined condition (e.g., conduct disorder or psychosis) is deemed off-label. There is a recognition that off-label prescribing does not imply improper or illegal use and is often necessary practice in children and adolescents in order to benefit the individual patient [13, 14]. Off-label prescribing may occur because the age of the child is lower than indicated on the license (often adults), due to indication, dose or route of administration [6, 7]. A recent scoping review by Meng et al. (2022) highlighted that antipsychotic agents were the most frequently studied medication to understand their off-label use [14, 15].

The antipsychotic most commonly prescribed to children and adolescents in our cohort was quetiapine (29%), followed closely by risperidone (28.6%) and aripiprazole (24.5%). In a study by Dinnissen and colleagues, who investigated adherence to antipsychotic prescribing guidelines among Dutch child and adolescent clinicians, the most prescribed antipsychotic was risperidone (68.3%), with aripiprazole (9.2%), olanzapine (6.7%) and quetiapine (1.6%) prescribed less frequently [15, 16]. A study by Radojčić and colleagues examined trends of antipsychotic prescribing to children and adolescents in England between 2000 and 2019, using primary care data [16, 17]. They reported the most frequently prescribed antipsychotics was risperidone (54.6%), followed by aripiprazole (17.6%), quetiapine (13.8%) and olanzapine (11.3%). The starting doses of antipsychotics prescribed in our dataset were aligned with the findings from the Dutch cohort [15, 16]. Radojčić et al., 2023 also reported that doses of antipsychotics in their English cohort were mostly within therapeutic ranges [16, 17].

The current study identified that antipsychotics were most commonly prescribed to target symptoms such as agitation and irritability rather than for a specific condition such as psychosis. The literature on indication for antipsychotic prescribing in children and adolescents is mixed. Some studies including those by Penfold et al. (2013) and Dinnissen et al. (2020) report findings similar to the current study [16, 18]. Radojčić et al. reported that indications associated with antipsychotics included ASD (12.7%), non-affective psychosis (8.6%), anxiety disorders (7.5%), ADHD (7.1%), depression (6.4%) and conduct disorders (6.1%) [16, 17]. 11.2% of prescriptions were associated with a non-specific mental health code and importantly, a limitation of the study identified by the authors was the inability to identify an indication in almost a third of prescriptions (Radojčić et al., 2023). A study by Rao and colleagues who surveyed child and adolescent psychiatrists in England on their prescribing patterns of atypical antipsychotics reported an indication of 8.6% in non-affective psychosis, 2.8% in affective psychosis, albeit 12.7% in autism spectrum disorder, , 7.1% in ADHD and 6.4% in depression [18, 19]. Similarly, in our study 12.4% of target conditions with an antipsychotic was for psychosis, albeit there was greater target symptom prescribing in this study which may highlight to a greater prevalence of prescribing based on target symptom clusters rather than defined disorder. Despite this, it is important to acknowledge that while off-label use cannot be labelled as poor clinical practice, the use of antipsychotic medication off-label may increase the likelihood of adverse events associated with antipsychotic use e.g., obesity, extrapyramidal side effects, hyperlipidaemia. Albeit there is mixed results of adverse events reported on off-label use in antipsychotic use in children or adolescents [20–22].

As expected, the main association of being prescribed an antipsychotic was by disorder in adjusted models. In our analysis, a higher number of co-morbid conditions were not associated with being prescribed an antipsychotic, which would be in contrast to similar adult studies ([22, 23]). Positively, in adjusted analysis, having a definitive diagnosis helped to illustrate a lower odds of being prescribed an antipsychotic (e.g., anxiety disorder). This in our view may highlight the importance of a clear diagnosis documented during the course of treatment to better understand the rationale of why a certain psychotropic was prescribed. In particular, as diagnostic changes or adjustments are frequent in a child and adolescent population and may provide greater clarity and transparency as to why a particular psychotropic was prescribed or changed longitudinally [23, 24].

The above discussion should be understood knowing the following limitations of this study. First, the available data does not capture if other specialists prescribed an antipsychotic (e.g., family doctors, neurologist, paediatricians), only child psychiatrists. The data was collected at source by paediatric psychiatry services and not trained data controllers, as such there may be input error. Conversely, this is a strength as services provided the information anonymously about service users and as such may have allowed greater transparency as to child psychiatrists prescribing for target symptoms and not definable disorders. The diagnosis classification used was not requested by services, albeit within the Irish context it is the International Classification of Disease–11 (ICD-11) or Diagnostic and statistical manual of mental disorders-5 (DSM-5). The data does not include other psychosocial interventions (i.e., non-pharmacological interventions); this is important to understand whether antipsychotics were prescribed in crisis (e.g., emotional dysregulation, aggression) or following unsuccessful non-pharmacological approaches. We did not have improved participant demographics (e.g., physical health conditions, family factors, socioeconomic factors and educational factors) which are important when considering child outcomes. With the available dataset, we were not able to determine whether these young people were drug naïve or if their prescriptions were repeated. This is deserving of further analysis using a different dataset. While the dose of medication was deemed to be maintenance daily dose, it is not possible with available data to determine if the dose was given pro necessitate rather than regular or daily. The prescribing standards were closed questions to determine if they had adhered to standard practice (e.g., monitoring), it did not provide explicit information (e.g., specific bloods or monitoring side-effects). Finally, we did not have available data on adverse reaction or if prescribing an antipsychotic alleviated the condition or symptoms.

In our study when clinicians in teams were asked to provide anonymously the rational/indication for antipsychotic use there was a high indication for target symptom cluster rather than a defined disorder or condition. This highlights the main implication which is transparency in indication documenting for clinicians and the prevalence of off-label prescribing. There is a need to understand if off-label prescribing has higher reported adverse reactions and adherence to monitoring standards for antipsychotic safety. In particular, it is surprising that quetiapine was the most prescribed antipsychotic in this study with known significant cardiometabolic adverse effects and little to no licensed indication in this age cohort. Similarly, a recent study highlighted that quetiapine was the most commonly prescribed antipsychotic by condition (e.g., psychosis) and risperidone by symptom (e.g., aggression) with a condition (e.g., autism spectrum disorder) [25]. These findings demonstrate a potential notable adherence gap between antipsychotic indication and guideline use. Furthermore, the need for a process to monitor psychotropic prescribing by dose and indication in Ireland is now necessary (e.g., national yearly audit or reporting system of prescriptions in CAMHS). Positively maintenance doses were low in this study which may be associated with a higher off-label use. This in turn may reflect a clinician’s hesitancy in increasing the dose and this is an area worthy of further research.

## Conclusion

To the best of our knowledge, this is the first study that describes the target conditions and target symptoms for off-label antipsychotic use in a paediatric psychiatry setting in Ireland. Our results show a high proportion of off-label (target symptom) prescribing with antipsychotic medication. There is a need to understand these findings cautiously within the complexity of child behaviour, co-morbidity and development.

## Supplementary Information

Below is the link to the electronic supplementary material.

Supplementary file 1

## Data Availability

The data controller is the Health Service Executive (HSE) (i.e., the national health service) in Ireland. The data may be accessed by application to the HSE and is pending explicit permission, data controller input and relevant ethical approval.
