# Supplementary material for: Antipsychotic prescribing: national findings of children and adolescents attending mental health services in Ireland
Source: Eur Child Adolesc Psychiatry. 2024 Apr 12;33(11):3861–70. doi: 10.1007/s00787-024-02428-4 (PMC11588832; doi:10.1007/s00787-024-02428-4)
Supplement: Supplementary file 1 [file 787_2024_2428_MOESM1_ESM.pdf]

Title: Antipsychotic prescribing: national findings of children and adolescents attending mental health services in Ireland.

David J O Driscoll[1], Suzanne McCarthy[2].

Supplementary Material

Figure S1: Flow chart of analysed participants

Figure S2: Target conditions of any antipsychotic medication (**A**), Risperidone only (**C**), Quetiapine only (**E**), Aripiprazole only (**G**), Olanzapine only (**I**), and target symptoms of any antipsychotic medication (**B**), Risperidone only (**D**), Quetiapine only (**F**), Aripiprazole only (**H**), Olanzapine only (**J**). See **Table 2** for (n) numbers of each medication.

Table S1: Description of questions and answers from the National Audit of prescribing in child and adolescent mental health service in Ireland.

Question	Allowed answers
**Is there a consultant child psychiatrist in place in your team?**	Yes or No
**Child gender**	Male, female, other
**Age on December 31st 2021**	In years
**Referral type to CAMHS**	Urgent, routine
**Date first seen in CAMHS**	DD/MM/YYYY
**Working diagnosis (tick if relevant):**	Moderate to severe anxiety disorder<br>Moderate to severe ADHD/ADD<br>Moderate to severe depression<br>Eating disorder<br>Obsessive Compulsive Disorder (OCD)<br>Psychotic illness<br>Bipolar affective disorder<br>Tics/Tourette's syndrome<br>Not specified/Not recorded
**If other working diagnosis,**	Please specific (free text)
**Is there evidence of consent obtained from parent/guardian(s) for prescribed medication?**	Yes or No
**Medication prescribed during audit time from 1st July 2021 and 31st December 2021**	Medication name (Generic or Brand name)<br>Target Condition<br>Target Symptoms<br>Date of commencement<br>Date of discontinuation<br>Starting daily dose (mcg, mg or g)<br>Maintenance daily dose (mcg, mg, g)
**Was medication prescribed by a consultant or in consultation with a consultant?**	Yes or No
**Was there documented evidence of baseline physical health assessment?**	Yes or No or Not applicable
**Was there documented evidence of physical health monitoring?**	Yes or No o Not applicable
**Was there documented evidence of communication with GP following medication initiation/review?**	Yes or No
**Was there a plan for follow up/review appointment recorded in the patient chart?**	Yes or No or Not applicable (i.e., Discharge)
**Please detail any further comments here**	Free text

Figures

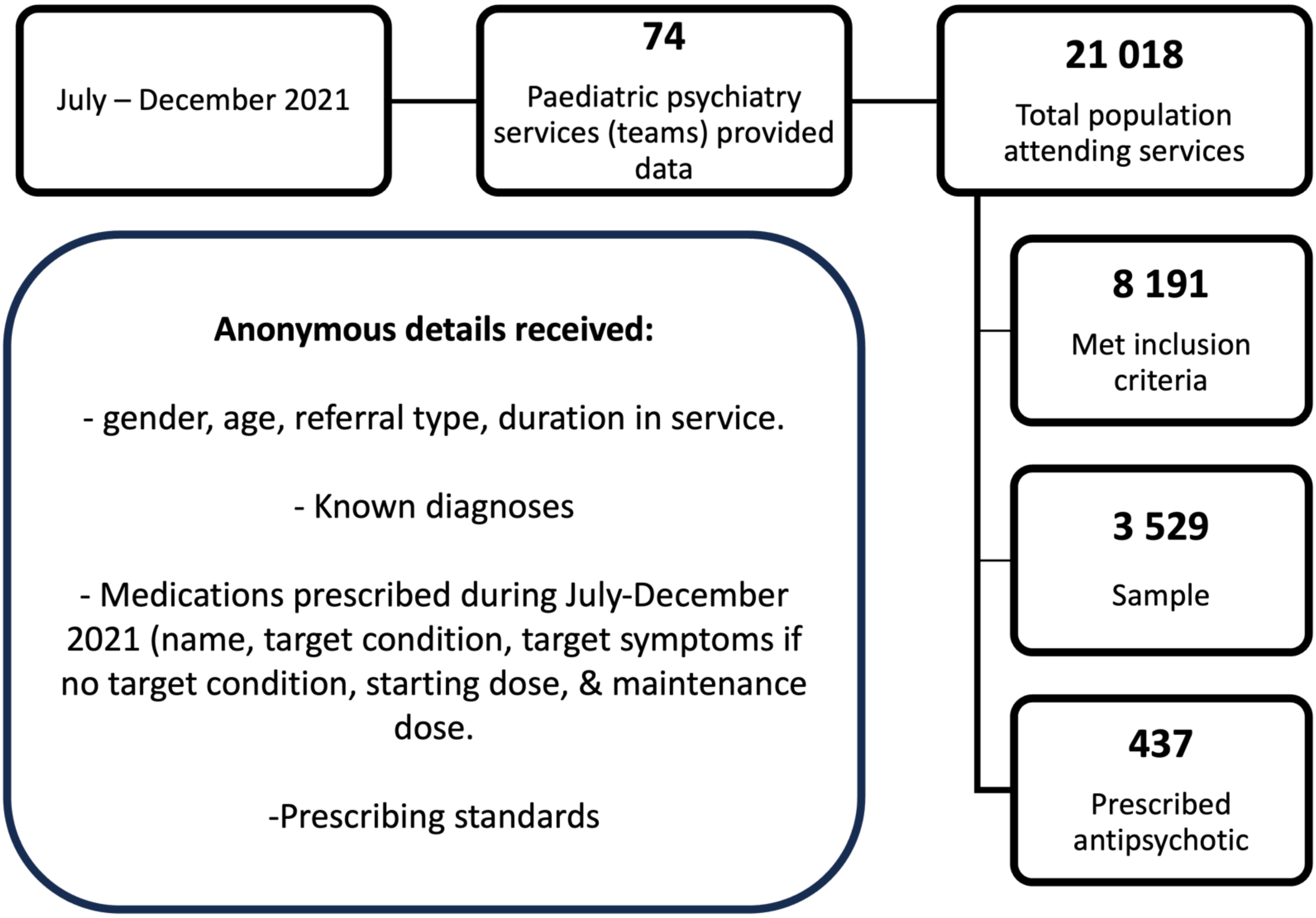

Figure S1: Flow chart of analysed participants. In 2021, the government of Ireland commissioned an audit team to investigate psychotropic prescribing in the child and adolescent mental health services in Ireland. This commission was due to a report 'Maskey Report' which was published in 2022 that highlighted several problems in the area of governance, clinical care and administrative practices on a specific CAMHS team. The Audit team contacted 74 child and adolescent mental health services (paediatric psychiatry services) to provide anonymised data on children or adolescents that were actively attending CAMHS between July 2021 and December 2021. This represents 21 018 children and adolescents attending the service nationally, 8 191 met the inclusion criteria (i.e., attending the service, 17 years of age or under, active attendance (i.e., attending appointments and/or receiving intervention) and prescribed any psychotropic medication during the above agreed timeline). The exclusion criteria included having a moderate to severe intellectual disability, primary diagnosis of autism spectrum disorder, and not meeting above inclusion criteria. Of the inclusion sample of 3 529, 437 were prescribed an antipsychotic.

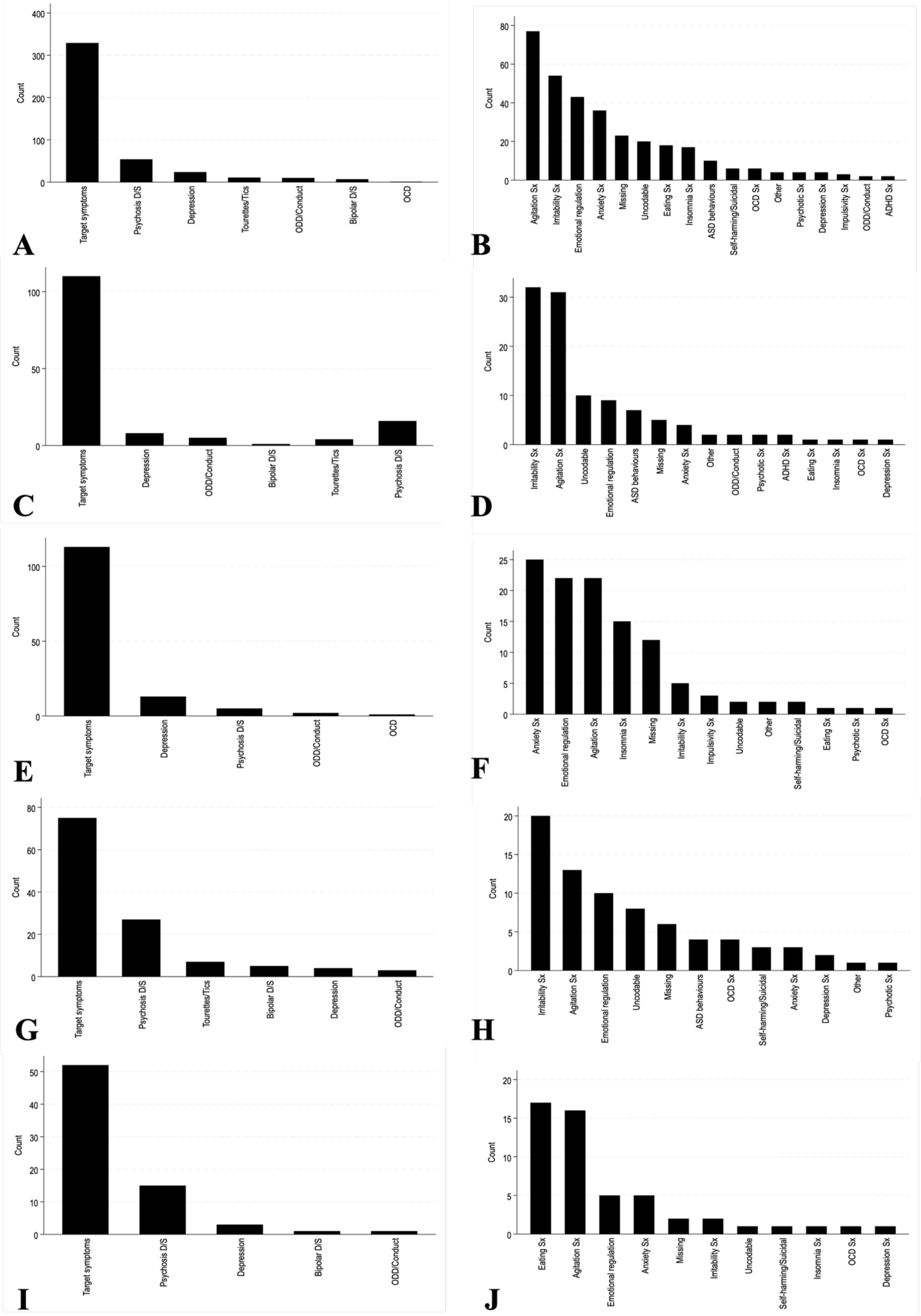

Figure S2: Target conditions of any antipsychotic medication (**A**), Risperidone only (**C**), Quetiapine only (**E**), Aripiprazole only (**G**), Olanzapine only (**I**), and target symptoms of any antipsychotic medication (**B**), Risperidone only (**D**), Quetiapine only (**F**), Aripiprazole only (**H**), Olanzapine only (**J**). See **Table 2** for (n) numbers of each medication.
